# Liganded Peroxisome Proliferator-Activated Receptors (PPARs) Preserve Nuclear Histone Deacetylase 5 Levels in Endothelin-Treated *Sprague-Dawley* Rat Cardiac Myocytes

**DOI:** 10.1371/journal.pone.0115258

**Published:** 2014-12-16

**Authors:** Haining Zhang, Zongjun Shao, Caroline P. Alibin, Crystal Acosta, Hope D. Anderson

**Affiliations:** 1 From the Canadian Centre for Agri-Food Research in Health and Medicine, St. Boniface General Hospital Research Centre, Winnipeg, Manitoba, Canada; 2 Department of Pharmacology & Therapeutics, Faculty of Medicine, University of Manitoba, Winnipeg, Manitoba, Canada; 3 College of Pharmacy, Faculty of Health Sciences, University of Manitoba, Winnipeg, Manitoba, Canada; Temple University, United States of America

## Abstract

Ligand activation of peroxisome proliferator-activated receptors (PPARs) prevents cardiac myocyte hypertrophy, and we previously reported that diacylglycerol kinase zeta (DGKζ) is critically involved. DGKζ is an intracellular lipid kinase that catalyzes phosphorylation of diacylglycerol; by attenuating DAG signaling, DGKζ suppresses protein kinase C (PKC) and G-protein signaling. Here, we investigated how PPAR-DGKζ signaling blocks activation of the hypertrophic gene program. We focused on export of histone deacetylase 5 (HDAC5) from the nucleus, a key event during hypertrophy, since crosstalk occurs between PPARs and other members of the HDAC family. Using cardiac myocytes isolated from Sprague-Dawley rats, we determined that liganded PPARs disrupt endothelin-1 (ET1)-induced nuclear export of HDAC5 in a manner that is dependent on DGKζ. When DGKζ-mediated PKC inhibition was circumvented using a constitutively-active PKCε mutant, PPARs failed to block ET1-induced nuclear retention of HDAC5. Liganded PPARs also prevented (i) activation of protein kinase D (the downstream effector of PKC), (ii) HDAC5 phosphorylation at 14-3-3 protein chaperone binding sites (serines 259 and 498), and (iii) physical interaction between HDAC5 and 14-3-3, all of which are consistent with blockade of nucleo-cytoplasmic shuttling of HDAC5. Finally, the ability of PPARs to prevent neutralization of HDAC5 activity was associated with transcriptional repression of hypertrophic genes. This occurred by first, reduced MEF2 transcriptional activity and second, augmented deacetylation of histone H3 associated with hypertrophic genes expressing brain natriuretic peptide, β-myosin heavy chain, skeletal muscle α-actin, and cardiac muscle α-actin. Our findings identify spatial regulation of HDAC5 as a target for liganded PPARs, and to our knowledge, are the first to describe a mechanistic role for nuclear DGKζ in cardiac myocytes. In conclusion, these results implicate modulation of HDAC5 as a mechanism by which liganded PPARs suppress the hypertrophic gene program.

## Introduction

Cardiac hypertrophy is the increase in myocardial mass provoked by hemodynamic stress or myocardial injury, and is a convergence point for many risk factors leading to heart failure. If left unchecked, prolonged hypertrophy is maladaptive and gives rise to cardiac arrest and/or failure [1], [2]. Thus, attenuation of hypertrophy is a promising therapeutic target to prevent heart failure.

At the cardiac myocyte level, hypertrophy is characterized by increases in cell size, protein synthesis, and changes in gene expression [3]. The latter includes sequential activation of immediate early response genes (e.g. proto-oncogenes such as c-fos and c-jun), a fetal gene program (e.g. atrial natriuretic peptide, skeletal muscle α-actin, and β-myosin heavy chain), and sarcomeric genes (e.g. cardiac muscle α-actin) [3]. In particular, re-induction of fetal genes such as brain natriuretic peptide (BNP) is one of the most consistent markers of transcriptional activation in hypertrophy [4].

Gene expression is regulated by the balance between histone acetyltransferase (HAT) and histone deacetylase (HDAC) activity. HATs promote transcription by acetylating nucleosomal histones to relax chromatin structure [5]. HDACs repress genes, at least in part, by deacetylating histones and condensing chromatin [6]. There are 4 families of HDACS (I, IIa, IIb, and IV) [7]. Class IIa HDACs are characterized by N-terminal extensions with conserved binding sites for transcription cofactors such as myocyte enhancer factor–2 (MEF2) [7]–[10]. They also contain 2 phosphorylation sites that, when phosphorylated by Ca^2+^/calmodu­lin-dependent protein kinase [9], promote binding of chaperone proteins that lead to nuclear export of HDACs, chromatin relaxation and de-repression of HDAC target genes [11]–[14]. In addition to chromatin deacetylation, repression of hypertrophy by class IIa HDACs [15]–[17] is achieved by binding MEF2, thereby inhibiting MEF2 activity [9], [11], [12]. In contrast, many hypertrophic stimuli promote phosphorylation and nuclear export of Class IIa HDACs. When HDACs are stimulated to dissociate from MEF2 and are then exported from the nucleus, MEF2 is free to promote hypertrophy through association with other pro-hypertrophic transcription factors such as GATA and NFAT [18]–[20], initiating hypertrophic transcription [17]. Accordingly, HDAC5-null mice develop exaggerated cardiac hypertrophy in response to pressure overload [21], whereas overexpression of HDAC5 suppresses MEF2-dependent transcription and agonist-dependent cardiac hypertrophy [17], [21].

It is therefore well-established that a key event in the hypertrophic gene response is phosphorylation of HDAC5 [11]. In particular, ET1 activates protein kinase C (PKC) and its downstream effector, protein kinase D (PKD). PKD in turn phosphorylates HDAC5 to initiate the signaling cascade which culminates in its exclusion from the nucleus [16]. Indeed, phosphorylation of HDAC5 promotes binding of 14-3-3 chaperone proteins, which then allows export of HDAC5 from the nucleus, de-repression of MEF2, and activation of fetal cardiac genes [12]. In addition, nuclear export of HDAC5 would allow histone acetylation and chromatin relaxation, thereby contributing to the activation of fetal genes [6].

Peroxisome proliferator-activated receptors (PPARs) have beneficial effects in the cardiovascular system. Activation of PPARs α [22]–[24], β/δ [24]–[27], and γ [22], [24], [28], [29] prevents fetal gene activation in response to mechanical or neurohumoral stimuli. A possible intersection between PPAR- and HDAC5-mediated repression of cardiac fetal genes lies in the ability of liganded PPARs to activate diacylglycerol kinase zeta (DGKζ) [24]. DGKs are intracellular lipid kinases that phosphorylate diacylglycerol (DAG) to produce phosphatidic acid. The resultant decrease in availability of DAG attenuates activation of PKC, thereby terminating an arm of pro-hypertrophic G protein signaling. We previously reported that DGKζ contributes to the ability of PPARs to suppress hypertrophy, but the mechanism by which fetal gene expression was attenuated remained unclear.

The aim of this study was to determine the mechanisms by which PPAR-DGKζ signaling inhibits hypertrophic gene expression. Because PKC/PKD activity opposes the anti-hypertrophic actions of HDAC5 [16], and liganded PPARs suppress myocyte hypertrophy by interfering with PKC signaling [24], we considered whether PPAR activation might preserve HDAC5 function. Then we sought to elucidate the mechanism by which liganded PPARs promote nuclear retention of HDAC5. Finally, we verified the role of DGKζ.

## Materials and Methods

### Materials

ET1, troglitazone, fenofibrate, GW501516, and antibodies against phospho-HDAC5 and β-actin were from Sigma-Aldrich (Oakville, Canada). DGKζ and 14-3-3 antibodies were from Santa Cruz Biotechnology (Santa Cruz, California). PKCε antibody was from Millipore (Temecula, California). HDAC5, PKD, and lamin B1 antibodies were from Cell Signaling Technology (Beverly, Massachusetts). Acetyl H3 antibody was from Actif Motif (Japan). R59022 was from EMD Biosciences, Inc. (Gibbstown, New Jersey). The PKCε kinase activity assay kit was Enzo Life Sciences (Brockville, Canada).

### Isolation of rat cardiac myocytes

The study is in full compliance with the Canadian Council on Animal Care, and approval was granted by the University of Manitoba Animal Care Committee (protocol 09-064). 1-day-old Sprague-Dawley rats were sacrificed by decapitation, ventricular myocytes were isolated by alternate cycles of 0.05% trypsin plus mechanical disruption as described [24], and cultured on gelatin-coated plates in DMEM containing 10% cosmic calf serum (CCS) (Hyclone) for 18–24 h prior to experimentation.

### Constitutively active PKCε construct

Constitutively active (ca)PKCε was constructed by first cloning rat PKCε cDNA into a pCDH lentivirus vector (MJS BioLynx Inc., Canada). The enzyme was then made constitutively-active by deletion of residues 154–163 of its inhibitory pseudosubstrate domain [30], and verified by DNA sequence analysis (University of Calgary Core DNA Services; Calgary, Canada).

### Lentiviral preparation and infection

Lentiviruses expressing small hairpin RNAs (shRNA) against DGKζ using shRNA-expressing plasmids (TRCN 0000025394, 0000025395, 0000025398; Open Biosystems; Ottawa, Canada) or caPKCε were prepared as previously described [24]. Scrambled sequences served as non-silencing controls. Lentivirus vector plasmids were co-transfected with psPAX2 (packaging) and pMD2.G (enveloping) vectors using FuGENE6 Reagent (Roche; Indianapolis, Indiana). High-titer lentiviral stock was produced in HEK-293T cells 48 h after transfection. Myocytes were infected for 24 h by application of the lentivirus to the culture medium, and then cultured for a further 72 h (to achieve shRNA knockdown or caPKCε expression) prior to treatments and further experimentation. Knockdown was confirmed by western blotting.

### Treatments

As applicable, myocytes were subjected to lentiviral infection. Myocytes were then rendered quiescent by serum deprivation (0.5% serum) for 24 h and pre-treated for 1 h with vehicle, troglitazone (1 µM), fenofibrate (10 µM), GW501516 (1 µM), or R59022 (10 µM).

Following the 1 h pre-treatment, hypertrophy was stimulated by addition of ET1 (0.1 µM).

### Cell Size

Myocyte size was assessed by immunofluorescent microscopy and computer-assisted planimetry, as previously described [31]. Each experiment consisted of 30 cells analyzed per experimental group.

### Isolation of cellular nuclear extracts and total protein quantification

Myocyte nuclear isolations were performed from 107 cells using a method adopted and modified from Cayman Chemical’s nuclear extraction kit (Ann Arbor, Michigan, USA, CN: 10009277). After treatments, myocytes were washed twice and suspended in phosphate-buffered saline (PBS) and centrifuged at 500×g for 5 minutes at 4°C. Cell pellets were re-suspended in a phosphatase inhibitor solution in PBS (20 mM NaF, 1 mM β-glycerophosphate, 1 mM Na3VO4). After centrifugation at 500×g for 5 minutes at 4°C, cell lysis was performed by incubation with hypotonic buffer (5 mM NaF, 10 µM Na2MoO4, 0.1 mM EDTA, 20 mM HEPES, pH 7.5) for 15 minutes on ice. Supernatants containing the cytosolic fraction were removed after the addition of 10% Igepal and subsequent centrifugation at 1000×g for 10 minutes at 4°C. The remaining pellets were re-suspended by vortex in extraction buffer (0.1 mM EDTA, 1.5 mM MgCl2, 420 mM NaCl, 20 mM NaF, 1 mM β-glycerophosphate, 10 mM Na3VO4, 25% glycerol, 0.5 mM phenylmethylsulfonyl fluoride, protease inhibitor tablets, 10 mM HEPES, pH 7.9) and rocked at 4°C for 15 minutes twice with mixing by vortex in between. Supernatants containing the nuclear fraction were collected by pelleting at 14, 000×g for 10 minutes at 4°C. Total protein content was quantified from the resulting nuclear extracts using the bicinchoninic acid (BCA) method.

### Diacylglycerol (DAG) assay

Nuclear DAG levels were quantified from myocyte nuclear isolates by competitive enzyme-linked immunosorbant assay (ELISA) using a Rat DAG ELISA kit (BlueGene, Shanghai, China, CN: E2D0010). In accordance with manufacturer’s instructions, 50 µg of total protein was incubated with DAG-HRP conjugate in a total volume of 160 µl in plates pre-coated with monoclonal anti-DAG for 1 hour at 37°C. Plates were washed five times and incubated with a proprietary HRP-substrate at 37°C for 15 minutes. After the addition of a stop solution the amount of DAG was measured at 450 nm.

### Immunoblotting and immunoprecipitation

Cell lysates were prepared in RIPA buffer and clarified by centrifugation. Conventional western blotting of cytosolic and nuclear extracts was used to assess levels of DGKζ, native and phosphorylated HDAC5, and native and phosphorylated PKD. To assess physical interaction between HDAC5 and 14-3-3 proteins, HDAC5 precipitates were generated from equal amounts of cardiac myocyte nuclear extracts, and 14-3-3 was detected by western blotting.

### DGKζ immunostaining

Cells were processed as previously described [32]. Briefly, myocytes were cultured in 4-chamber slides, serum-deprived, and then subjected to the indicated treatments. Cells were then washed with PBS and fixed with 3.7% paraformaldehyde, followed by PBS containing 0.2% Triton X-100 for 2 min. Slides were blocked with PBS containing 0.2% bovine serum albumin and normal horse IgG for 1 h. Nuclei were stained with DAPI (blue; Invitrogen, Grand Island, NY), and immunofluorescent staining was performed using a primary DGKζ antibody followed by a secondary antibody conjugated to Alexa-488 (green). Cells were imaged by fluorescence microscopy.

### PKCε activity

PKCε was immunoprecipitated from equal amounts of cardiac myocyte nuclear extracts. PKCε activity was assessed using a commercially-available ELISA-based PKC activity assay kit according to the manufacturer’s protocol.

### MEF2 luciferase assay

Myocytes were infected with lentivirus that expresses a MEF2-luciferase reporter (i.e. firefly luciferase reporter gene driven by a basic promoter element joined to tandem repeats of MEF2; SABiosciences). Myocytes were maintained in DMEM/10% CSC for 24 h, then serum-deprived for 24 h. Following treatments (as described in figure legends), luciferase activity was measured from lysates using a Luciferase Reporter Assay System (Promega).

### Hypertrophic gene acetylation status

Cells were cross-linked with 1% formaldehyde, collected and lysed. Soluble chromatin was fragmented by partial digestion with micrococcal nuclease (NEB), and proteins were immunoprecipitated using an anti-acetylated histone H3 antibody. Following reversal of protein-DNA cross-linking, the DNA was purified by phenol/chloroform extraction and used for quantitative PCR with primers specific for the gene promoters of brain natriuretic peptide (BNP; 5′-CCGGACACCCAGCCCCAGGATA-3′ and 5′-GTCTCGCCTCCGCAAGCAGCTC-3′), β-myosin heavy chain (5′-TGGTGGAAGGCGGCGTACAGGT-3′ and 5′-CGCCTCCAGCCGCTCCTTCTC-3′), skeletal muscle α-actin (5′-GTCCAGCCCAGCCCTTCAGCAG-3′ and 5′-CCGGACCGGGCCGTATATGGAG-3′) or cardiac muscle α-actin (5′-GTCTGGGAGCCCCCTGGCTGAT-3′ and 5′-TGGACGGGGTCAGTTGGAGCAG-3′). Aliquots of chromatin obtained before immunoprecipitation were also analyzed (input control). Results are expressed as the relative amount of PCR product aliquots immunoprecipitated with acetylated H3, normalized by the input control.

### Statistics

n-Values are indicated in legends. Error bars represent SEMs. Data were subjected to unpaired t-test or one-way ANOVA and, as appropriate, post-hoc analyses (Newman–Keuls, Bonferroni, or Holm-Sidak multiple comparison tests).

## Results

### DGK contributes to the anti-hypertrophic actions of PPARs

**Figure 1:**
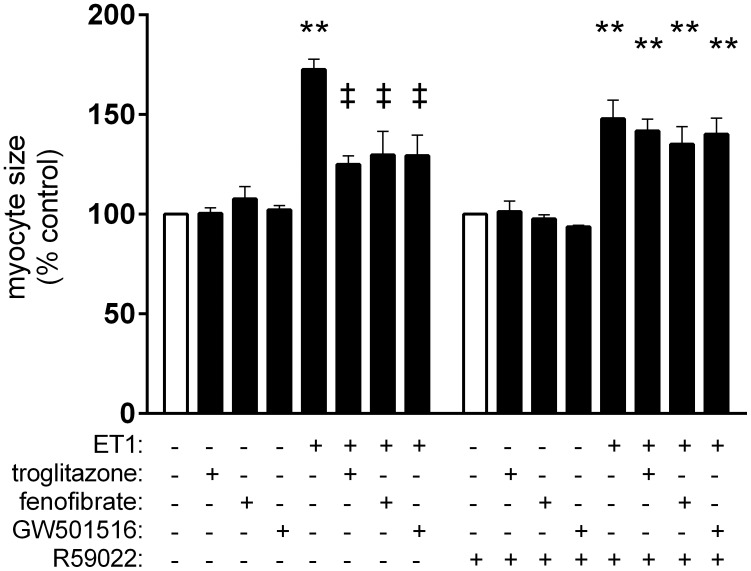
DKG inhibition blocks PPAR-dependent suppression of ET1-induced cardiac myocyte hypertrophy. Myocytes deprived of serum for 24 h were stimulated with ET1 (0.1 µM). Subsets of cells were also pre-treated with vehicle or PPAR ligands (troglitazone, 1 µM; fenofibrate, 10 µM; GW501516, 1 µM; 1 h). Cell surface areas of individual cells were quantified as described in “Methods,” and presented as percent of myocyte size (µm^2^) vs. vehicle-treated controls. n = 4–5 sets of 30 cells analyzed. **p<0.01 vs. vehicle-treated controls. ‡p<0.01 vs. ET1-treated cells.

We first verified the effects of liganded PPARs, and the role of DGK, using the DGK inhibitor, R59022. ET1 increased myocyte size, and this was attenuated by troglitazone, fenofibrate, and GW501516. The anti-hypertrophic actions of PPAR ligands were abolished by R59022 (Fig. 1).

### Liganded PPARs preserve nuclear HDAC5 levels in a manner dependent on DGKζ activity and PKCε attenuation

**Figure 2:**
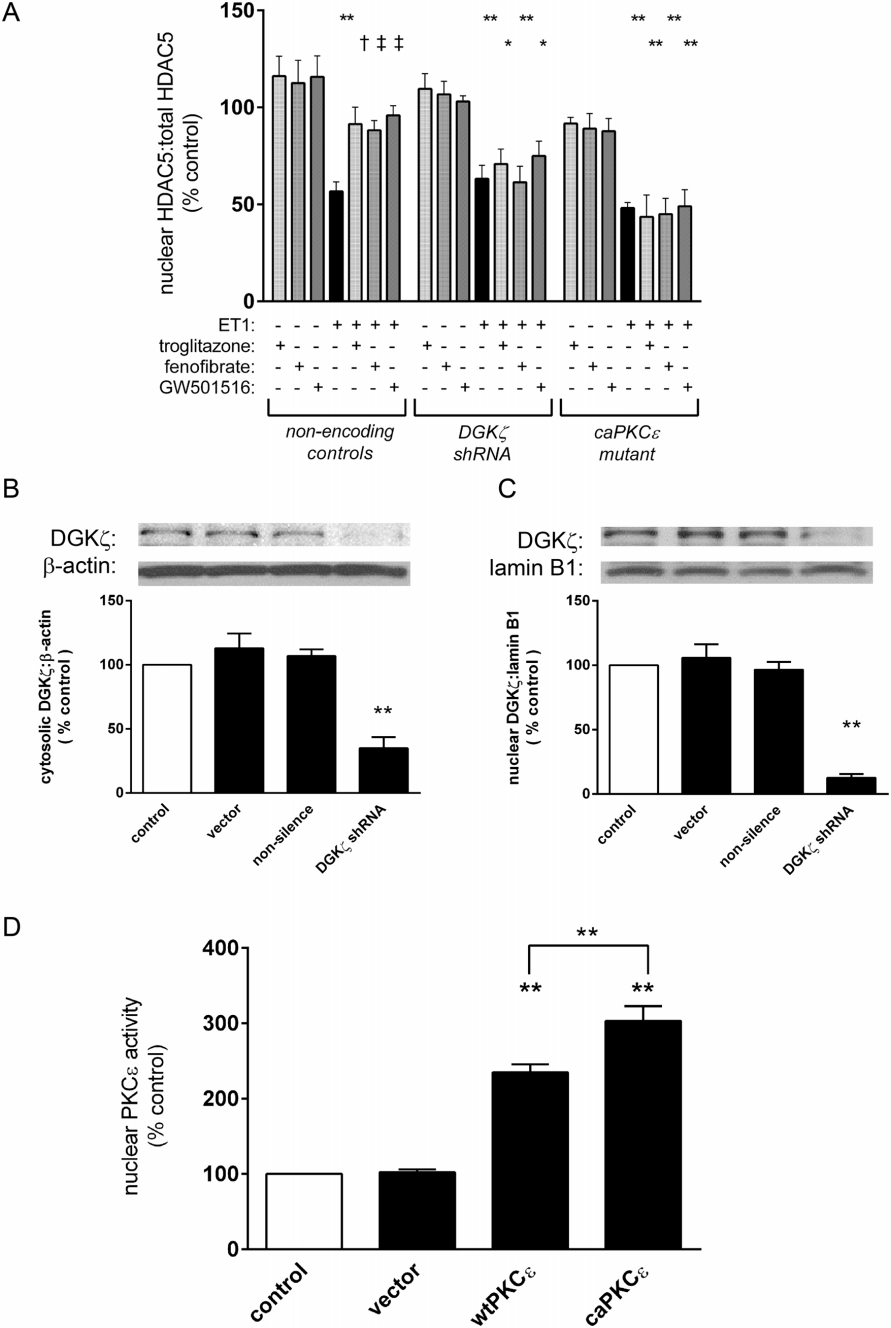
Modulation of nuclear HDAC5 by liganded PPARs. Myocytes deprived of serum for 24 h were stimulated with ET1 (0.1 µM). Subsets of cells were also pre-treated with vehicle or PPAR ligands (troglitazone, 1 µM; fenofibrate, 10 µM; GW501516, 1 µM; 1 h), shRNA knockdown of DGKζ, or expression of a constitutively active (ca) PKCε mutant. *A,* ET1 treatment significantly reduced nuclear HDAC5, and this was attenuated by liganded PPARs. *B/C,* DGKζ knockdown or *D*, circumvention of DGKζ-mediated PKC inhibition using caPKCε blocked PPAR-dependent retention of HDAC5 in the nucleus (Figure 2A). n = 4. *p<0.05 and **p<0.01 vs. controls. †p<0.05 and ‡p<0.01 vs. ET1-treated cells.

ET-1 (0.1 µM; 24 h) reduced HDAC5 content in nuclear extracts (57±5% vs. control, p<0.01), and this was attenuated by troglitazone (1 µM), fenofibrate (10 µM), and GW501516 (1 µM) (Fig. 2A). RNA silencing of DGKζ (Fig. 2B/2C) or expression of a lentiviral-driven caPKCε construct (Fig. 2D) abolished the ability of PPARs to promote nuclear retention of HDAC5 (Fig. 2A).

### Liganded PPARs preserve nuclear DGKζ levels

**Figure 3:**
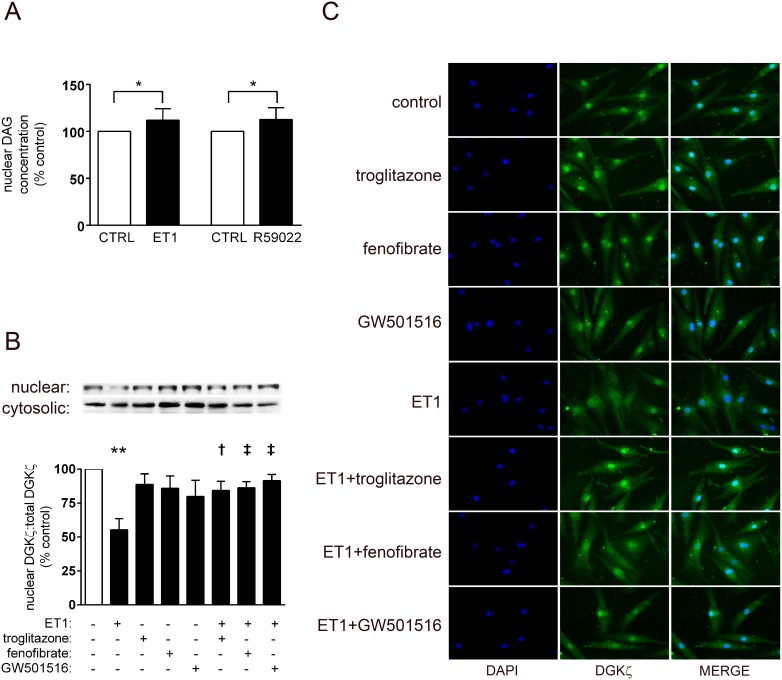
Modulation of nuclear DGKζ by liganded PPARs. Myocytes deprived of serum for 24 h were stimulated with ET1 (0.1 µM). Subsets of cells were also pre-treated with vehicle, R59022 (DGK inhibitor, 10 µM), or PPAR ligands (troglitazone, 1 µM; fenofibrate, 10 µM; GW501516, 1 µM; 1 h). *A,* Nuclear DAG levels were assessed by ELISA of myocyte nuclear isolates. As would be expected as an output of GPCR-signaling, ET1 increased nuclear DAG levels. Likewise, inhibition of DGK using R59022 (i.e. inhibition of phosphorylative conversion of DAG to phosphatidic acid) also led to augmented nuclear DAG concentrations. n = 4–6. To determine how ET1 might be modulating nuclear DAG concentrations, nuclear DGKζ protein was assessed by *B,* western blotting of nuclear extracts and total lysates, and *C*, immunostaining and is presented as percent of normalized DGKζ protein vs. vehicle-treated controls. The ability of ET1 to reduce nuclear DGKζ was abolished by liganded PPARs. n = 4. *p<0.05 and **p<0.01 vs. vehicle-treated controls. †p<0.05 and ‡p<0.01 vs. ET1-treated cells.

Our previous report of PPAR-DGKζ crosstalk [24], [31] extends to the cardiac myocyte nucleus. We first determined that ET1 (0.1 µM; 24 h) increased nuclear DAG levels; likewise, inhibition of DGKζ using R59022 led to greater nuclear DAG concentrations (Fig. 3A). Consistent with this, ET1 reduced DGKζ content in nuclear extracts (55±8% vs. control, p<0.01), and this was attenuated by troglitazone, fenofibrate, and GW501516 (Figs. 3B and 3C).

### Liganded PPARs attenuate nuclear PKCε in a manner dependent on DGKζ

**Figure 4:**
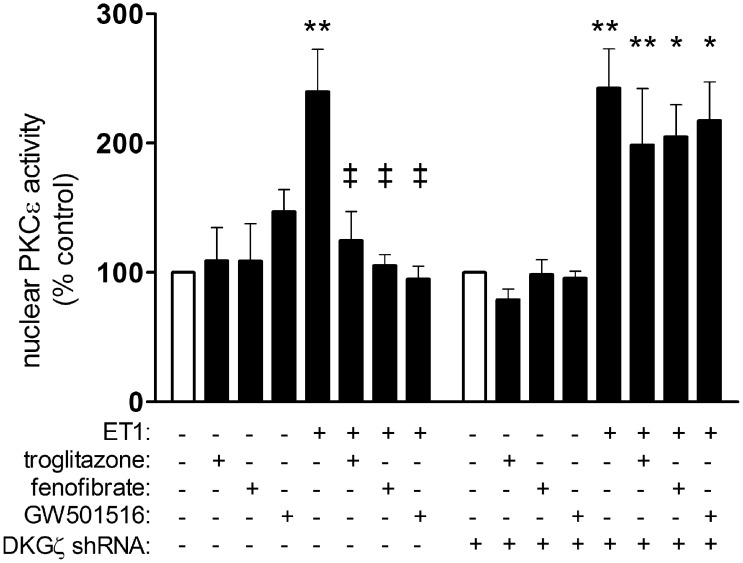
Liganded PPARs inhibit ET1-dependent activation of nuclear PKCε via DGKζ . Myocytes deprived of serum for 24 h were stimulated with ET1 (0.1 µM). Subsets of cells were also pre-treated with vehicle or PPAR (troglitazone, 1 µM; fenofibrate, 10 µM; GW501516, 1 µM; 1 h). Activity of PKCε immunoprecipitates was assessed using a commercially-available ELISA-based PKC activity assay kit. The ability of PPARs to attenuate ET1-induced PKCε activity in the nucleus was abolished by shRNA knockdown of DGKζ. n = 3. *p<0.05 and **p<0.01 vs. vehicle-treated controls. ‡p<0.01 vs. ET1-treated cells.

As the ability of PPARs to preserve nuclear HDAC5 levels relies on DGKζ activity and suppression of PKCε activity (Fig. 2), we determined that PPAR-DGKζ signaling attenuates nuclear PKCε activity. ET1 increased PKCε activity in nuclear extracts (240±8% vs. control, p<0.01), and this was attenuated by troglitazone, fenofibrate, and GW501516 (Fig. 4). However, the ability of liganded PPARs to prevent PKCε activation was abolished by shRNA knockdown of DGKζ.

### HDAC5 phosphorylation and activation are attenuated by liganded PPARs

**Figure 5:**
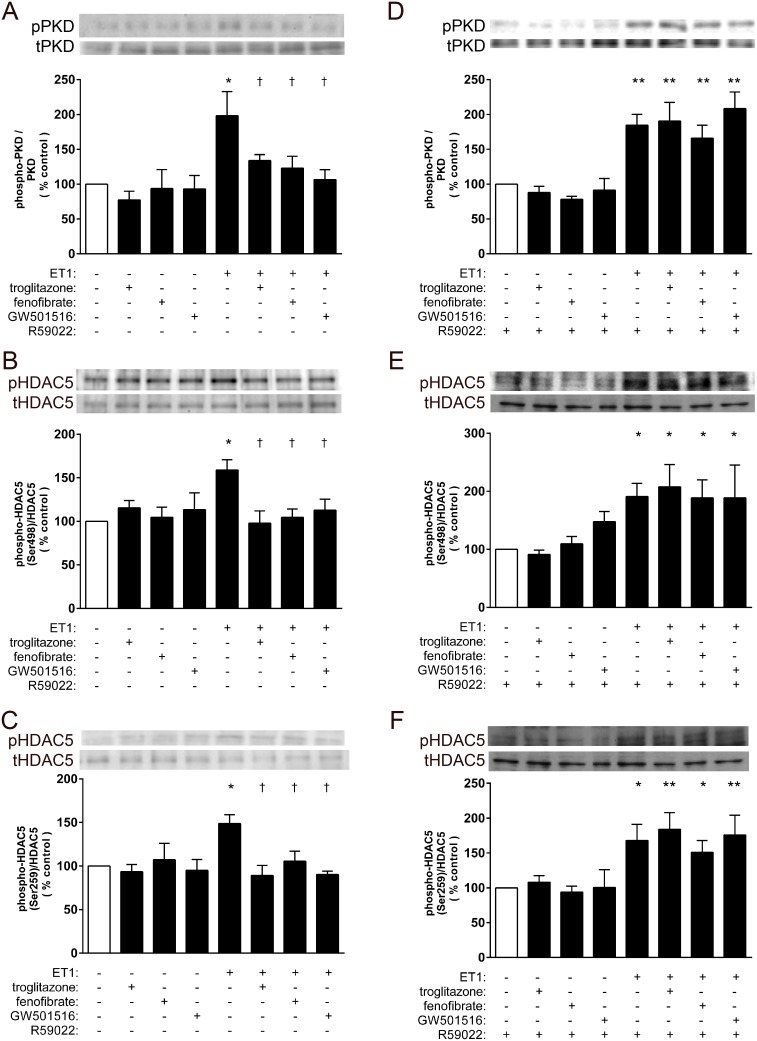
Modulation of PKD and HDAC5 phosphorylation by liganded PPARs. Myocytes deprived of serum for 24 h were stimulated with ET1 (0.1 µM). Subsets of cells were also pre-treated with vehicle or PPAR ligands (troglitazone, 1 µM; fenofibrate, 10 µM; GW501516, 1 µM; 1 h) as well as R59022 (DGK inhibitor, 10 µM). ET1 treatment significantly induced phosphorylative activation of *A*, PKD (Ser744/748) and HDAC5 (*B*, Ser498 and *C*, Ser259). PKD and HDAC5 phosphorylation was attenuated by liganded PPARs, whereas R59022 abolished PPAR effects. n≥3. *p<0.05 and **p<0.01 vs. controls. †p<0.05 and ‡p<0.01 vs. ET1-treated cells.

ET1 induced activation of PKD, the downstream effector of PKC known to directly phosphorylate HDAC5 [16], as evidenced by phosphorylation at Ser744/748 (Fig. 5A). Phosphorylation of HDAC5 [11] initiates the signaling cascade which results in nuclear export of HDAC5 [16]. ET1 also promoted phosphorylation of HDAC5 at the highly conserved 14-3-3 binding sites: Ser498 (Fig. 5B) and Ser259 (Fig. 5C). PPAR ligands prevented PKD and HDAC5 phosphorylation in a manner sensitive to reversal by R59022 (Figs. 5D–4F).

### Liganded PPARs attenuate physical interaction between HDAC5 and 14-3-3 chaperone proteins

**Figure 6:**
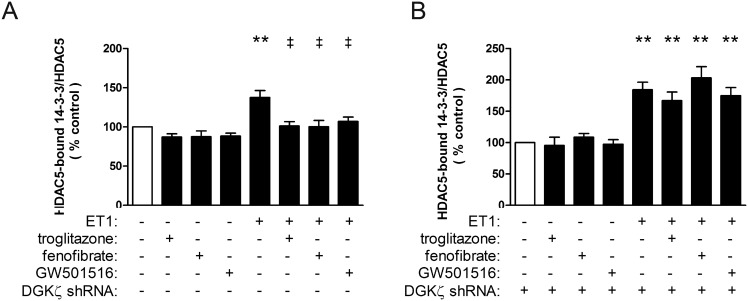
Interaction of nuclear HDAC5 with 14-3-3 is modulated by liganded PPARs. Myocytes deprived of serum for 24 h were stimulated with ET1 (0.1 µM). Subsets of cells were also pre-treated with vehicle or PPAR ligands (troglitazone, 1 µM; fenofibrate, 10 µM; GW501516, 1 µM; 1 h) in the presence and absence of shRNA knockdown of DGKζ. HDAC5 was immunoprecipitated, and interaction between HDAC5 and 14-3-3 was assessed biochemically by immunoblotting. Data are presented as percent of normalized 14-3-3 vs. vehicle-treated controls. ET1 treatment significantly increased binding between HDAC5 and 14-3-3. n = 4. The ability of ET1 to increase HDAC5•14-3-3 interaction was *A,* abolished by liganded PPARs (n = 6), and *B*, restored by shRNA knockdown of DGKζ (n = 4). **p<0.01 vs. vehicle-treated controls. ‡p<0.01 vs. ET1-treated cells.

Binding of 14-3-3 chaperone proteins facilitates phosphorylation and export of HDAC5 from the nucleus and subsequent de-repression of pro-hypertrophic genes [12]. Accordingly, ET1 increased binding between HDAC5 and 14-3-3 (138±9% vs. control, p<0.01), and this was attenuated by troglitazone, fenofibrate, and GW501516 (Fig. 6). However, the ability of liganded PPARs to prevent HDAC5•14-3-3 binding was abolished by shRNA knockdown of DGKζ.

### Suppression of MEF2 by liganded PPARs involves DGKζ

**Figure 7:**
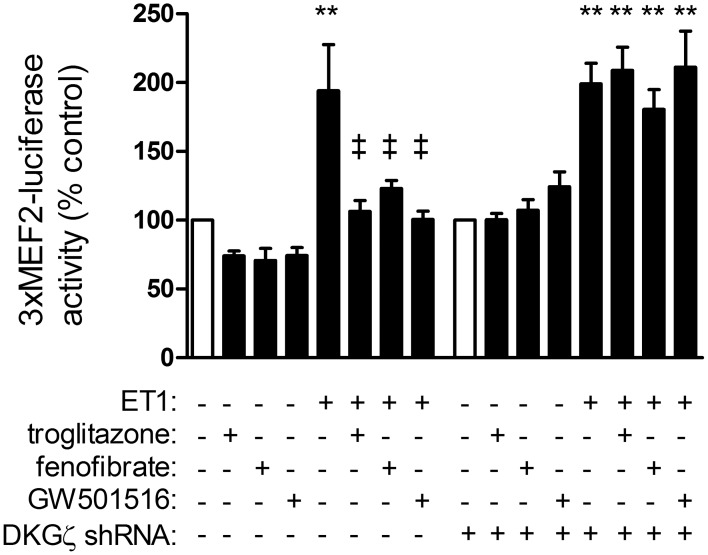
Liganded PPARs inhibit ET1-dependent activation of MEF2 via DGKζ. Myocytes were infected with a lentivirus that expresses a MEF2-luciferase reporter, deprived of serum for 24 h, then stimulated with ET1 (0.1 µM). Subsets of cells were also pre-treated with vehicle or PPAR (troglitazone, 1 µM; fenofibrate, 10 µM; GW501516, 1 µM; 1 h). Luciferase activity was measured from lysates using a Luciferase Reporter Assay System (Promega). The ability of PPARs to attenuate ET1-induced MEF2 activity was abolished by shRNA knockdown of DGKζ. n = 5–6. **p<0.01 vs. vehicle-treated controls. ‡p<0.01 vs. ET1-treated.

Repression of fetal cardiac genes by HDAC5 is achieved, at least in part, through association with MEF2 [17]. In the absence of nuclear HDAC5, MEF2 is free to promote transcription of hypertrophic genes. We found that ET1 increased MEF2 transcriptional activity, as shown by activation of a 3xMEF2-luciferase reporter (Fig. 7; 194±33% vs. control, p<0.01), and this was prevented by PPAR ligands. However, the ability of liganded PPARs to prevent MEF2 activation was abolished by shRNA knockdown of DGKζ.

### Liganded PPARs suppress histone H3 acetylation by a DGKζ-dependent mechanism

Acetylation of histone H3 is a response to hypertrophic stimuli *in vitro* [33] and *in vivo* [34]. Indeed, ET1 induced H3 acetylation of hypertrophic genes, which favors their transcription (Table 1). Liganded PPARs suppressed ET1-dependent histone acetylation, and again, these PPAR actions were abolished by shRNA knockdown of DGK*ζ*.

**Table 1:** Effect of DGKζ knockdown on PPAR-mediated suppression of histone H3 acetylation of hypertrophic genes.

hypertrophicgene	shRNA	ET1 (% control)	troglitazone (% control)	troglitazone + ET1 (% control)	fenofibrate (% control)	fenofibrate + ET1 (% control)	GW501516 (% control)	GW501516+ ET1 (% control)
brain natriuretic peptide	**non-silence**	450±70%**	**160±60%^‡^**	**210±60%^‡^**	**190±20%^‡^**	**190±70%‡**	**160±20%^‡^**	**190±50%^‡^**
	DGKζ	370±80%^**^	130±10%^‡^	300±50%^**^	140±20%^‡^	370±60%^**^	150±50%^‡^	380±80%^**^
β-myosin heavy chain	**non-silence**	440±50%**	**170±30%^‡^**	**170±50%^‡^**	**190±70%^‡^**	**220±80%^‡^**	**220±60%^‡^**	**210±20%^‡^**
	DGKζ	310±70%^**^	140±50%^‡^	280±70%^**^	150±10%^‡^	310±80%^**^	120±0%^‡^	250±40%*
skeletal muscle α-actin	**non-silence**	400±70%**	**120±20%^‡^**	**170±20%^‡^**	**150±90%^‡^**	**170±60%^‡^**	**160±3070%^‡^**	**220±40%** * **^,‡^**
	DGKζ	440±30%^**^	120±40%^‡^	410±50%^**^	90±20%^‡^	490±80%^**^	110±10%^‡^	360±50%**
cardiac muscle α-actin	**non-silence**	520±10%**	**150±60%^‡^**	**170±70%^‡^**	**160±40%^‡^**	**230±20%^‡^**	**140±50%^‡^**	**190±40%^‡^**
	DGKζ	300±50%^**^	170±40%†	250±50%*	180±20%†	330±50%^**^	130±30%^‡^	240±70%*

mean ± SEM; n = 3;

*p<0.05 and *p<0.01 vs control;

† p<0.05 & ^‡^p<0.01 vs ET1.

## Discussion

**Figure 8:**
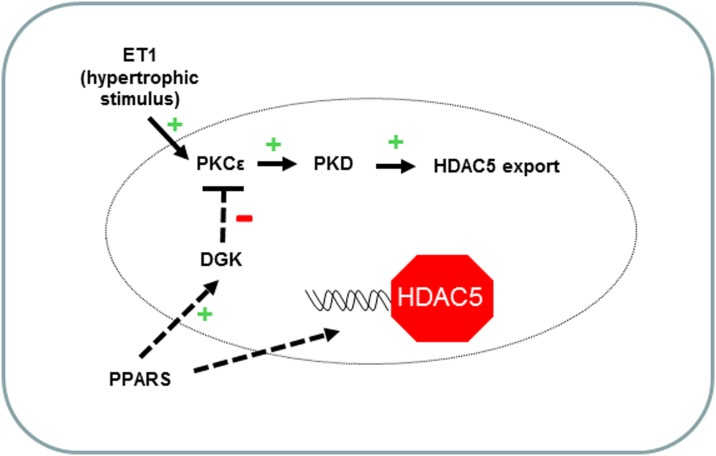
Proposed mechanism by which liganded PPARs abrogate ET1-induced hypertrophic gene expression. ET1 induces accumulation of DAG in the plasma membrane, which causes translocation and activation of PKCε, an early signaling mediator of hypertrophy. Downstream events including phosphorylation of PKD and HDAC5 give rise to 14-3-3-facilitated export of HDAC5 from the nucleus and subsequently de-repression of hypertrophic genes. Liganded PPARs stimulate nuclear DGKζ to phosphorylate DAG. The resulting decreased availability of DAG attenuates PKCε and downstream signaling effectors, resulting in nuclear retention of HDAC5.

These findings provide insight into the mechanisms by which liganded PPARs suppress hypertrophic gene activation in cardiac myocytes. As depicted in Fig. 8, liganded PPARs increase nuclear DGKζ, which in turn prevents ET1-induced activation of nuclear PKCε and PKD, phosphorylation of HDAC5 at conserved 14-3-3-binding sites, and association between HDAC5 and 14-3-3. Collectively, these events lead to nuclear retention of HDAC5. Rescue of HDAC5 activity in the nucleus favors repression of hypertrophic genes vis-à-vis suppression of MEF2 transcriptional activity and augmented deacetylation of histone H3 associated with hypertrophic genes.

Our finding that liganded PPARs increase nuclear DGKζ levels may be attributable to either their inhibitory effects on PKCε or their function as nuclear receptor transcription factors [35]. Topham *et al*. reported that PKC regulates the nuclear localization of DGKζ via phosphorylation of the MARCKS-homology domain, a motif which is similar to some nuclear localization sequences. However, this regulatory mechanism was restricted to only the PKC α and γ isoforms [36]. It is more likely, therefore, that PPARs modulate nuclear DGKζ levels by influencing transcription of the DGKζ gene, either directly or indirectly. Admittedly, the PPRE consensus sequence does not appear in the DGKζ gene promoter, but this does not preclude direct binding of PPARs. Nakachi *et al*. detected 167 gene promoters with strict PPARγ binding during adipocyte differentiation in 3T3-L1 cells, yet PPRE consensus sequences were present in only 15 of these target genes [37]. Three of these genes were activated by PPARγ, lack consensus PPREs, and instead contain imperfect or atypical PPREs that vary in the binding site and/or spacer sequences [38]. Indirect regulation of the DGKζ promoter might also be achieved through the formation of a complex with other transcription factors such as Sp1. The DGKζ promoter contains Sp1 sites, and PPAR-dependent modulation of numerous genes involves Sp1 [39]–[42]. In fact, we have data to support the notion that PPAR-mediated regulation of the DGKζ promoter occurs through a complex comprised of PPAR and Sp1 (see S1 Figure). Troglitazone-induced DGKζ expression was blocked by mithramycin, an Sp1 inhibitor [39]. Also, troglitazone promoted binding of Sp1 to the DGKζ promoter and promoted binding between PPARγ and Sp1. These data suggest that PPARs and Sp1 are recruited to the DGKζ promoter, perhaps as a PPAR•Sp1-containing complex. Whatever the proximal signaling mechanism, the mechanism by which liganded PPAR modulate nuclear DGKζ, at least in part vis-à-vis the nature of the putative interaction between PPARs and the DGKζ promoter, requires further study.

DGKζ overexpression suppresses cardiac hypertrophy through inhibition of PKCε [43] [44]. Here, we extend this by demonstrating that, specifically in the nucleus, PPARs terminate PKCε and its downstream effector PKD [16] via DGKζ. PKD phosphorylates HDAC5 at serines 259 and 498, which serve as docking sites for 14-3-3 [12]; in turn, 14-3-3 binding masks the nuclear localization sequence situated between the phosphorylation sites and reveals a nuclear export sequence at the C terminus. The end result is HDAC5 export from the nucleus, chromatin relaxation and de-repression of HDAC target hypertrophic genes [9], [11]–[14]. Thus, the ability of PPAR/DGKζ signaling to terminate nuclear PKCε/PKD activity serves to rescue HDAC5 function, thereby conferring resistance to ET1 as a hypertrophic stimulus. These findings are consistent with a previous report that mutation of serines 259 and 498 results in loss 14-3-3 protein binding and a significant increase in HDAC5 deacetylation activity [45].

Our notion, that preserved HDAC function is responsible for PPAR effects, is consistent with a previous report in which PPARγ-induced adipogenesis was attenuated by a chemical inhibitor of class II HDACs, MC1568 [46]. Furthermore, using PPRE-luciferase-expressing reporter mice, Nebbioso *et al* showed that MC1568, impairs PPARγ signaling mostly in heart and adipose tissues [46]. In hypertrophied myocytes, our findings suggest liganded PPARs suppress hypertrophic gene expression, at least in part, by tethering of MEF2 to HDAC5. This inhibits MEF2 transcriptional activity (Fig. 7) by abolishing binding to other pro-hypertrophic transcription factors such as GATA and nuclear factor of activated T-cells (NFAT) [18]–[20].

In addition to MEF2 sequestration, our data suggest a secondary anti-hypertrophic action of HDAC5. In myocytes, induction of fetal genes such as the atrial natriuretic peptide (ANP) and β-myosin heavy chain genes is associated with acetylation of histone H3 [17], [47] that is preferentially localized to the promoter regions of the gene [33]. Further, histone H3 acetylation is exaggerated in the hearts of pressure overloaded mice [34]. We found that PPARs suppress acetylation of histone H3 on the promoters of genes encoding BNP, β-myosin heavy chain, skeletal muscle α-actin, and cardiac muscle α-actin (Table 1), suggesting that histone H3 deacetylation may be a secondary mechanism by which nuclear PPAR/DGKζ signaling represses the hypertrophic gene program.

Other signaling mechanisms within the nucleus may also contribute to PPAR-induced repression of hypertrophic genes. For example, PPARs might disrupt HAT activity. Work by others suggests that the anti-hypertrophic actions of PPARα are related to suppression of AP-1 DNA-binding activity [23]. ET1-induced PKCε leads to activation of the AP-1 transcription factor [43], [48], and forced overexpression of DGKζ in cardiac myocytes blocks not only ET1-dependent hypertrophy, but also AP-1 signaling [43]. AP-1 is regulated by p300, a transcriptional co-activator and HAT. In fact, p300-induced acetylation of dimeric AP-1 complexes enhances AP-1 binding to DNA [49]. Thus, pro-hypertrophic ET1/PKCε-dependent activation of AP-1 signaling might also be attenuated by PPAR-DGKζ crosstalk and depletion of DAG, though this remains to be determined.

The present study shows for the first time that the ability of PPAR ligands to block activation of hypertrophic genes in response to ET1 involves PPAR/DGKζ crosstalk in the cardiac myocyte nucleus. The mechanistic contribution of DGKζ is likely due to attenuation of PKCε signaling by depleting its substrate, DAG. Retention of HDAC5 in the nucleus appears to be a consequence of attenuated PKCε and PKD signaling, leading to suppression of pro-hypertrophic events such as histone acetylation of hypertrophic genes and MEF2 activation. Taken in context with other studies on DGKζ, interventions that target PPAR/DGKζ signaling might represent a novel therapeutic approach to address the problem of cardiac hypertrophy.

## Supporting Information

S1 Figure **Troglitazone promotes recruitment of PPARγ and Sp1 to the DGKζ promoter.**
(PDF)

## References

[1] Levy D, Garrison RJ, Savage DD, Kannel WB, Castelli WP (1990) Prognostic implications of echocardiographically determined left ventricular mass in the Framingham Heart Study. N Engl J Med 322:1561–1566. 2139921 10.1056/NEJM199005313222203

[2] Ho KK, Pinsky JL, Kannel WB, Levy D (1993) The epidemiology of heart failure: the Framingham Study. J Am Coll Cardiol 22:6A–13A. 8376698 10.1016/0735-1097(93)90455-a

[3] Kuwahara K, Nishikimi T, Nakao K (2012) Transcriptional regulation of the fetal cardiac gene program. J Pharmacol Sci 119:198–203. 22786561 10.1254/jphs.12r04cp

[4] LaPointe MC (2005) Molecular regulation of the brain natriuretic peptide gene. Peptides 26:944–956. 15911064 10.1016/j.peptides.2004.08.028

[5] Jenuwein T, Allis CD (2001) Translating the histone code. Science 293:1074–1080. 11498575 10.1126/science.1063127

[6] Mathiyalagan P, Keating ST, Du XJ, El-Osta A (2014) Chromatin modifications remodel cardiac gene expression. Cardiovasc Res 103:7–16. 24812277 10.1093/cvr/cvu122

[7] Haberland M, Montgomery RL, Olson EN (2009) The many roles of histone deacetylases in development and physiology: implications for disease and therapy. Nat Rev Genet 10:32–42. 19065135 10.1038/nrg2485 PMC3215088

[8] Grozinger CM, Hassig CA, Schreiber SL (1999) Three proteins define a class of human histone deacetylases related to yeast Hda1p. Proc Natl Acad Sci U S A 96:4868–4873. 10220385 10.1073/pnas.96.9.4868 PMC21783

[9] Lu J, McKinsey TA, Nicol RL, Olson EN (2000) Signal-dependent activation of the MEF2 transcription factor by dissociation from histone deacetylases. Proc Natl Acad Sci U S A 97:4070–4075. 10737771 10.1073/pnas.080064097 PMC18151

[10] Miska EA, Karlsson C, Langley E, Nielsen SJ, Pines J, et al (1999) HDAC4 deacetylase associates with and represses the MEF2 transcription factor. Embo J 18:5099–5107. 10487761 10.1093/emboj/18.18.5099 PMC1171580

[11] McKinsey TA, Zhang CL, Lu J, Olson EN (2000) Signal-dependent nuclear export of a histone deacetylase regulates muscle differentiation. Nature 408:106–111. 11081517 10.1038/35040593 PMC4459600

[12] McKinsey TA, Zhang CL, Olson EN (2000) Activation of the myocyte enhancer factor-2 transcription factor by calcium/calmodulin-dependent protein kinase-stimulated binding of 14-3-3 to histone deacetylase 5. Proc Natl Acad Sci U S A 97:14400–14405. 11114197 10.1073/pnas.260501497 PMC18930

[13] Grozinger CM, Schreiber SL (2000) Regulation of histone deacetylase 4 and 5 and transcriptional activity by 14-3-3-dependent cellular localization. Proc Natl Acad Sci U S A 97:7835–7840. 10869435 10.1073/pnas.140199597 PMC16631

[14] McKinsey TA, Zhang CL, Olson EN (2001) Identification of a signal-responsive nuclear export sequence in class II histone deacetylases. Mol Cell Biol 21:6312–6321. 11509672 10.1128/MCB.21.18.6312-6321.2001 PMC87361

[15] Backs J, Song K, Bezprozvannaya S, Chang S, Olson EN (2006) CaM kinase II selectively signals to histone deacetylase 4 during cardiomyocyte hypertrophy. J Clin Invest 116:1853–1864. 16767219 10.1172/JCI27438 PMC1474817

[16] Vega RB, Harrison BC, Meadows E, Roberts CR, Papst PJ, et al (2004) Protein kinases C and D mediate agonist-dependent cardiac hypertrophy through nuclear export of histone deacetylase 5. Mol Cell Biol 24:8374–8385. 15367659 10.1128/MCB.24.19.8374-8385.2004 PMC516754

[17] Zhang CL, McKinsey TA, Chang S, Antos CL, Hill JA, et al (2002) Class II histone deacetylases act as signal-responsive repressors of cardiac hypertrophy. Cell 110:479–488. 12202037 10.1016/s0092-8674(02)00861-9 PMC4459650

[18] Molkentin JD, Lu JR, Antos CL, Markham B, Richardson J, et al (1998) A calcineurin-dependent transcriptional pathway for cardiac hypertrophy. Cell 93:215–228. 9568714 10.1016/s0092-8674(00)81573-1 PMC4459646

[19] Morin S, Charron F, Robitaille L, Nemer M (2000) GATA-dependent recruitment of MEF2 proteins to target promoters. Embo J 19:2046–2055. 10790371 10.1093/emboj/19.9.2046 PMC305697

[20] Youn HD, Chatila TA, Liu JO (2000) Integration of calcineurin and MEF2 signals by the coactivator p300 during T-cell apoptosis. Embo J 19:4323–4331. 10944115 10.1093/emboj/19.16.4323 PMC302027

[21] Chang S, McKinsey TA, Zhang CL, Richardson JA, Hill JA, et al (2004) Histone deacetylases 5 and 9 govern responsiveness of the heart to a subset of stress signals and play redundant roles in heart development. Mol Cell Biol 24:8467–8476. 15367668 10.1128/MCB.24.19.8467-8476.2004 PMC516756

[22] Liang F, Wang F, Zhang S, Gardner DG (2003) Peroxisome proliferator activated receptor (PPAR)alpha agonists inhibit hypertrophy of neonatal rat cardiac myocytes. Endocrinology 144:4187–4194. 12933694 10.1210/en.2002-0217

[23] Irukayama-Tomobe Y, Miyauchi T, Sakai S, Kasuya Y, Ogata T, et al (2004) Endothelin-1-induced cardiac hypertrophy is inhibited by activation of peroxisome proliferator-activated receptor-alpha partly via blockade of c-Jun NH2-terminal kinase pathway. Circulation 109:904–910. 14967736 10.1161/01.CIR.0000112596.06954.00

[24] Huang Y, Zhang H, Shao Z, O’Hara KA, Kopilas MA, et al (2011) Suppression of endothelin-1-induced cardiac myocyte hypertrophy by PPAR agonists: role of diacylglycerol kinase zeta. Cardiovasc Res 90:267–275. 21183507 10.1093/cvr/cvq401

[25] Planavila A, Rodriguez-Calvo R, Jove M, Michalik L, Wahli W, et al (2005) Peroxisome proliferator-activated receptor beta/delta activation inhibits hypertrophy in neonatal rat cardiomyocytes. Cardiovasc Res 65:832–841. 15721863 10.1016/j.cardiores.2004.11.011

[26] Sheng L, Ye P, Liu YX, Han CG, Zhang ZY (2008) Peroxisome proliferator-activated receptor beta/delta activation improves angiotensin II-induced cardiac hypertrophy in vitro. Clin Exp Hypertens 30:109–119. 18293166 10.1080/10641960801945840

[27] Lee KS, Park JH, Lee S, Lim HJ, Park HY (2009) PPARdelta activation inhibits angiotensin II induced cardiomyocyte hypertrophy by suppressing intracellular Ca2+ signaling pathway. J Cell Biochem 106:823–834. 19224536 10.1002/jcb.22038

[28] Yamamoto K, Ohki R, Lee RT, Ikeda U, Shimada K (2001) Peroxisome proliferator-activated receptor gamma activators inhibit cardiac hypertrophy in cardiac myocytes. Circulation 104:1670–1675. 11581147 10.1161/hc4001.097186

[29] Asakawa M, Takano H, Nagai T, Uozumi H, Hasegawa H, et al (2002) Peroxisome proliferator-activated receptor gamma plays a critical role in inhibition of cardiac hypertrophy in vitro and in vivo. Circulation 105:1240–1246. 11889020 10.1161/hc1002.105225

[30] Wotton D, Ways DK, Parker PJ, Owen MJ (1993) Activity of both Raf and Ras is necessary for activation of transcription of the human T cell receptor beta gene by protein kinase C, Ras plays multiple roles. J Biol Chem 268:17975–17982. 8349679

[31] Alibin CP, Kopilas MA, Anderson HD (2008) Suppression of cardiac myocyte hypertrophy by conjugated linoleic acid: role of peroxisome proliferator-activated receptors alpha and gamma. J Biol Chem 283:10707–10715. 18283099 10.1074/jbc.M800035200

[32] Anderson HD, Wang F, Gardner DG (2004) Role of the epidermal growth factor receptor in signaling strain-dependent activation of the brain natriuretic peptide gene. J Biol Chem 279:9287–9297. 14645255 10.1074/jbc.M309227200

[33] Kaneda R, Ueno S, Yamashita Y, Choi YL, Koinuma K, et al (2005) Genome-wide screening for target regions of histone deacetylases in cardiomyocytes. Circ Res 97:210–218. 16002748 10.1161/01.RES.0000176028.18423.07

[34] Kong Y, Tannous P, Lu G, Berenji K, Rothermel BA, et al (2006) Suppression of class I and II histone deacetylases blunts pressure-overload cardiac hypertrophy. Circulation 113:2579–2588. 16735673 10.1161/CIRCULATIONAHA.106.625467 PMC4105979

[35] Lee KC, Lee Kraus W (2001) Nuclear receptors, coactivators and chromatin: new approaches, new insights. Trends Endocrinol Metab 12:191–197. 11397643 10.1016/s1043-2760(01)00392-7

[36] Topham MK, Bunting M, Zimmerman GA, McIntyre TM, Blackshear PJ, et al (1998) Protein kinase C regulates the nuclear localization of diacylglycerol kinase-zeta. Nature 394:697–700. 9716136 10.1038/29337

[37] Nakachi Y, Yagi K, Nikaido I, Bono H, Tonouchi M, et al (2008) Identification of novel PPARgamma target genes by integrated analysis of ChIP-on-chip and microarray expression data during adipocyte differentiation. Biochem Biophys Res Commun 372:362–366. 18489901 10.1016/j.bbrc.2008.05.037

[38] Gervois P, Chopin-Delannoy S, Fadel A, Dubois G, Kosykh V, et al (1999) Fibrates increase human REV-ERBalpha expression in liver via a novel peroxisome proliferator-activated receptor response element. Mol Endocrinol 13:400–409. 10076997 10.1210/mend.13.3.0248

[39] Deng T, Shan S, Li PP, Shen ZF, Lu XP, et al (2006) Peroxisome proliferator-activated receptor-gamma transcriptionally up-regulates hormone-sensitive lipase via the involvement of specificity protein-1. Endocrinology 147:875–884. 16269451 10.1210/en.2005-0623

[40] Chung SS, Choi HH, Cho YM, Lee HK, Park KS (2006) Sp1 mediates repression of the resistin gene by PPARgamma agonists in 3T3-L1 adipocytes. Biochem Biophys Res Commun 348:253–258. 16876120 10.1016/j.bbrc.2006.07.048

[41] Bonofiglio D, Qi H, Gabriele S, Catalano S, Aquila S, et al (2008) Peroxisome proliferator-activated receptor gamma inhibits follicular and anaplastic thyroid carcinoma cells growth by upregulating p21Cip1/WAF1 gene in a Sp1-dependent manner. Endocr Relat Cancer 15:545–557. 18509005 10.1677/ERC-07-0272

[42] Bonofiglio D, Gabriele S, Aquila S, Qi H, Belmonte M, et al (2009) Peroxisome proliferator-activated receptor gamma activates fas ligand gene promoter inducing apoptosis in human breast cancer cells. Breast Cancer Res Treat 113:423–434. 18293083 10.1007/s10549-008-9944-1

[43] Takahashi H, Takeishi Y, Seidler T, Arimoto T, Akiyama H, et al (2005) Adenovirus-mediated overexpression of diacylglycerol kinase-zeta inhibits endothelin-1-induced cardiomyocyte hypertrophy. Circulation 111:1510–1516. 15781737 10.1161/01.CIR.0000159339.00703.22

[44] Arimoto T, Takeishi Y, Takahashi H, Shishido T, Niizeki T, et al (2006) Cardiac-specific overexpression of diacylglycerol kinase zeta prevents Gq protein-coupled receptor agonist-induced cardiac hypertrophy in transgenic mice. Circulation 113:60–66. 16380548 10.1161/CIRCULATIONAHA.105.560771

[45] Greco TM, Yu F, Guise AJ, Cristea IM (2011) Nuclear import of histone deacetylase 5 by requisite nuclear localization signal phosphorylation. Mol Cell Proteomics 10: M110 004317. 10.1074/mcp.M110.004317 PMC3033682 21081666

[46] Nebbioso A, Dell’Aversana C, Bugge A, Sarno R, Valente S, et al (2010) HDACs class II-selective inhibition alters nuclear receptor-dependent differentiation. J Mol Endocrinol 45:219–228. 20639404 10.1677/JME-10-0043

[47] Kuwahara K, Saito Y, Ogawa E, Takahashi N, Nakagawa Y, et al (2001) The neuron-restrictive silencer element-neuron-restrictive silencer factor system regulates basal and endothelin 1-inducible atrial natriuretic peptide gene expression in ventricular myocytes. Mol Cell Biol 21:2085–2097. 11238943 10.1128/MCB.21.6.2085-2097.2001 PMC86819

[48] Takahashi H, Takeishi Y, Miyamoto T, Shishido T, Arimoto T, et al (2004) Protein kinase C and extracellular signal regulated kinase are involved in cardiac hypertrophy of rats with progressive renal injury. Eur J Clin Invest 34:85–93. 14764070 10.1111/j.1365-2362.2004.01304.x

[49] Wang WM, Wu SY, Lee AY, Chiang CM (2011) Binding site specificity and factor redundancy in activator protein-1-driven human papillomavirus chromatin-dependent transcription. J Biol Chem 286:40974–40986. 21937452 10.1074/jbc.M111.290874 PMC3220474

