# Supplementary material for: Liganded Peroxisome Proliferator-Activated Receptors (PPARs) Preserve Nuclear Histone Deacetylase 5 Levels in Endothelin-Treated Sprague-Dawley Rat Cardiac Myocytes
Source: PLoS One. 2014 Dec 16;9(12):e115258. doi: 10.1371/journal.pone.0115258 (PMC4267838; doi:10.1371/journal.pone.0115258)
Supplement: S1 Figure — Troglitazone promotes recruitment of PPARγ and Sp1 to the DGKζ promoter. (PDF) [file pone.0115258.s001.pdf]

Figure S1: Troglitazone promotes recruitment of PPARγ and Sp1 to the DGKζ promoter

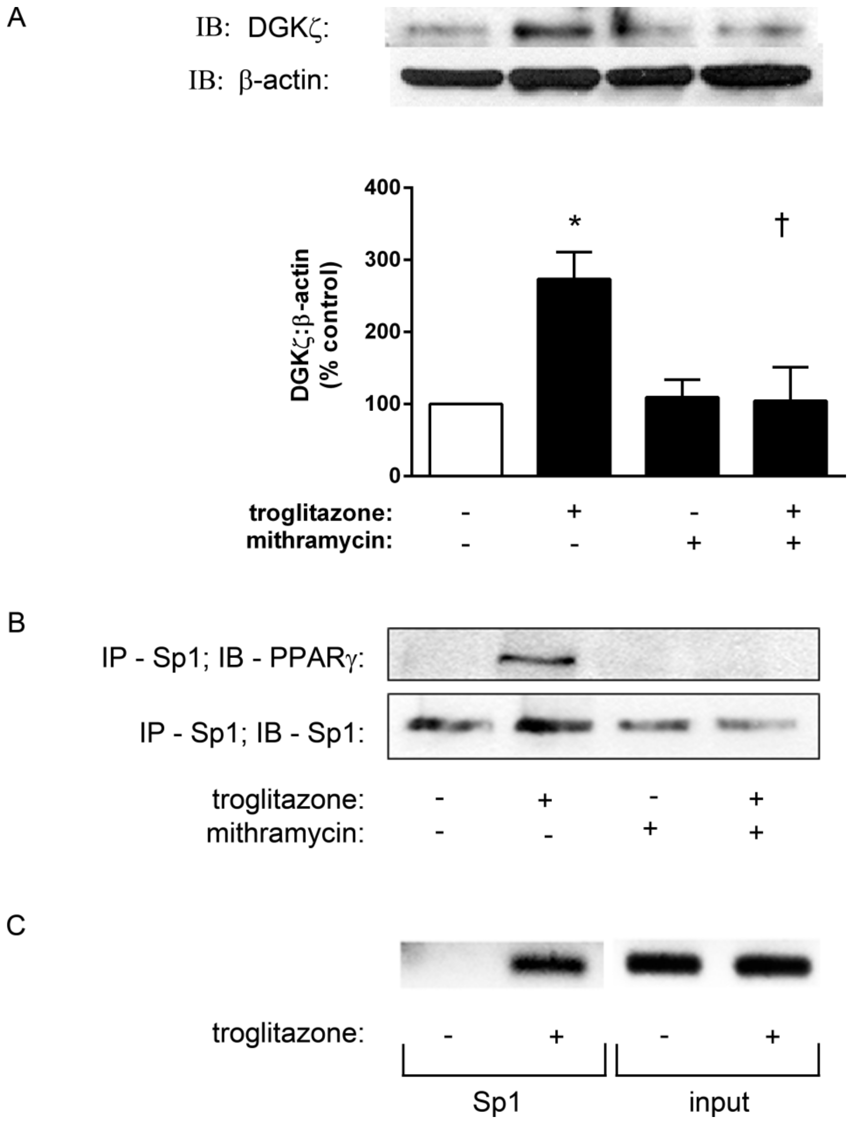

Legend: In response to troglitazone, PPARγ and Sp1 are recruited to the DGKζ promoter, perhaps as a PPARγ•Sp1 complex. Neonatal rat cardiac myocytes were treated with vehicle or troglitazone (PPARγ agonist; 10^{-6} mol/L) in the presence and absence of mithramycin (Sp1 inhibitor; 10^{-7} mol/L). *A*, DGKζ protein was assessed by western blotting and presented as percent of normalized protein vs. vehicle-treated controls. n=3. *p<0.05 *vs.* vehicle-treated control. †p<0.05 vs. troglitazone alone. *B*, Representative blots of co-immunoprecipitation analysis showing that troglitazone leads to physical interaction between PPARγ and Sp1. *C*, ChIP assay showing that troglitazone promotes Sp1 interaction with a DGKζ promoter fragment (Primers used to amplify the DGKζ promoter fragment were as follows: primer1: 5'-GGT ACC GGA GCG GAG GCT GCT TC-3', primer2: 5'- CTC GAG TGC AGG AGG GTT AGG AGC TGA CC-3').
